# High glucose induces renal tubular epithelial injury via Sirt1/NF-kappaB/microR-29/Keap1 signal pathway

**DOI:** 10.1186/s12967-015-0710-y

**Published:** 2015-11-09

**Authors:** Ling Zhou, De-yu Xu, Wen-gang Sha, Lei Shen, Guo-yuan Lu, Xia Yin, Ming-jun Wang

**Affiliations:** Department of Nephrology, The First Affiliated Hospital of Soochow University, Suzhou, 215006 China; Department of Endocrinology, The First Affiliated Hospital of Soochow University, Suzhou, 215006 China; Department of Rheumatology, The First Affiliated Hospital of Soochow University, 188 shizi Rd., Suzhou, 215006 People’s Republic of China

**Keywords:** Acetyl-p65, HK-2, miR-29, Nrf2, Serum creatinine

## Abstract

**Objective:**

Diabetic nephropathy (DN) is a serious complication that commonly confronted by diabetic patients. A common theory for the pathogenesis of this renal dysfunction in diabetes is cell injury, inflammation as well as oxidative stress. In this content, the detailed molecular mechanism underlying high glucose induced renal tubular epithelial injury was elaborated.

**Methods:**

An in vivo rat model of diabetes by injecting streptozotocin (STZ) and an in vitro high glucose incubated renal tubular epithelial cell (HK-2) model were used. Expression levels of Keap1, nuclear Nrf2 and p65 were determined by western blotting. Level of microR-29 (miR-29) was assessed using quantitative RT-PCR. Combination of p65 and miR-29 promotor was assessed using chromatin immunoprecipitation. Keap1 3′-UTR activity was detected using luciferase reporter gene assay. Cell viability was determined using MTT assay.

**Results:**

In diabetic rat, miR-29 was downregulated and its expression is negatively correlated with both of serum creatinine and creatinine clearance. In high glucose incubated HK-2 cell, deacetylases activity of Sirt1 was attenuated that leads to decreased activity of nuclear factor kappa B (NF-κB). NF-κB was demonstrated to regulate miR-29 expression by directly binding to its promotor. The data of luciferase assay showed that miR-29 directly targets to Keap1 mRNA. While high glucose induced down regulation of miR-29 contributed to enhancement of Keap1 expression that finally reduced Nrf2 content by ubiquitinating Nrf2. Additionally, overexpression of miR-29 effectively relieved high glucose-reduced cell viability.

**Conclusion:**

High glucose induces renal tubular epithelial injury via Sirt1/NF-κB/microR-29/Keap1 signal pathway.

**Electronic supplementary material:**

The online version of this article (doi:10.1186/s12967-015-0710-y) contains supplementary material, which is available to authorized users.

## Background

Diabetes mellitus is a common metabolic disorder which is associated with chronic complications such as angiopathy, retinopathy, and peripheral neuropathy. It was recognized that diabetes also can lead to nephropathy. Since then, studies in experimental models and in patients observed structural abnormalities in vascular and glomerular [1]. However, tubular cells are proved to be primary targets of hyperglycemia which is the major cause for renal injury and previous evidence indicated that chronic exposure to elevated blood glucose levels contributes to the organic pathologic changes in clinical diabetes nephropathy (DN) [2, 3]. Thus searching for therapy targets might be benefit for concurrency of DN.

In previous study, high glucose induced inflammatory response as well as activated inflammation related signal pathway. Sirt1, a class III histone deacetylase, is recognized as an important regulator in many high glucose-related inflammatory diseases [4]. For example, Sirt1 activator blunting pro-inflammatory pathways in mice fed a high fat, high calorie diet [5]. Especially, Sirt1 and its modulation of the acetylation status of the p65 subunit of nuclear factor kappa B (NF-κB) play an important role in regulating the inflammatory and apoptotic responses in cells or tissues [6]. As an essential nuclear transcription factor, NF-κB is commonly activated by wide variety of cell-tress stimuli including obesity, oxidative stress, as well as stimulated by hyperglycemia. Accumulated evidence pointed out that NF-κB was upregulated in diabetic rat kidneys [7] and its inhibition contributed to significant amelioration of DN [8]. Although observation has showed that master regulator of inflammation of NF-κB is essential for pathological process of DN, the detailed downstream intracellular signal transduction is not yet clear.

MicroRNAs are small non-coding RNAs and function as a key regulatory that affect protein expression by process of post-transcription. Abnormal expression levels of microRNAs have been described in DN and altered protein expression that influence renal function [9]. Among these, miR-29 is observed in diabetic patients [10] and also ameliorates hyperglycemia-induced renal dysfunction [11]. Mechanisms of alteration of microRNA expression focused on controlling transcriptional activity by transcription factor binding to promotor. Notably, decreased expression of miR-29 by NF-κB signal has been described in cells and tissues [12, 13]. Thus we speculated that NF-κB/miR-29 axis might be involved in high glucose incubated renal cell.

MicroRNAs regulate gene expression via suppressing translation or causing mRNA degradation. We attempted to figure out the target gene for miR-29. The transcription factor Nrf2 (NF-E2–related factor 2) has been postulated to be a key regulator of anti-cell press genes and loss of Nrf2 activity leads to alteration of ingredient of urinary in streptozotocin (STZ)-induced diabetic mice [14]. The controller of Nrf2 activity is Keap1 (Kelch-like ECH–associated protein), an adaptor protein for Cullin3-based ubiquitin E3 ligase by ubiquitination and subsequent degradation of Nrf2 [15]. Accumulated evidence revealed that Keap1 was controlled by microRNAs, such as miR-200, miR-141 and miR-28 [16]. We made a prediction of the possible binding site in Keap1 3′ UTR by miR-29 and this was demonstrated by online bioinformatics analysis (data is not shown).

Taken together, in this content, we established in vivo rat model of diabetes and in vitro high glucose incubated renal tubular cell and investigated the Sirt1/NF-κB/miR-29/Keap1/Nrf2 signal pathway in process of high glucose induced renal tubular injury.

## Methods

### Animal of model of acute diabetes

Adult male Wistar rats (200–250 g) used for the present study were obtained from Experimental Animal Institute of Soochow University. The animals were housed in a controlled environment with standard conditions of temperature and humidity. The illumination was kept in a cycle of an alternating 12 h light and dark. The animal protocols were approved by the animal ethics committee of Suzhou University, Suzhou, China (No. 49, 17th Nov 2000).

Rat model of diabetes was induced by intraperitoneal injection of streptozotocin (STZ; 65 mg/kg body weight) (Sigma-Aldrich Co., St. Louis, MO, USA) in 0.1 mol/L citrate buffer. The rats in normal group were received citrate buffer. The blood glucose was examined on day 3 and 5 after STZ treatment and successful diabetic model was with a standard of rat with greater than 16.7 mM blood glucose in both examinations. Serum was collected at 0, 4, 8, 12, 16 weeks for evaluation of blood index. Renal tubular was obtained for detection of miR-29 level.

### Sample collection and tissue preparation

During the experimental period, the animals were housed individually in metabolic cages due to different time periods (0, 4, 8, 12 and 16 weeks). The blood samples were collected from caudal vein over a separately period. Serum was obtained by centrifugation for 5 min at 4000 rpm centrifugalization and maintained at −80 °C. After blood collection, the rats were sacrificed by using local ether anaesthesia. Blood was removed from kidney by rat perfusion with normal saline through ascending aorta. Then the left kidney was rapidly removed and renal tubular was separated for homogenate preparation. Nuclear and cytoplasmic fractions were extracted from renal tissues using a relevant extract kit (Thermo Fisher Scientific, Waltham, MA, USA) following the manufacturer’s instructions and were used for following molecular assays.

### Assessment of serum creatinine and BUN

Plasma creatinine (Creatinine Assay Kit; Abcam, USA) and BUN (Urea Nitrogen (BUN) Colorimetric Detection Kit; Arbor Assays, USA) levels were measured according to manufacturer’s instructions.

### Cell culture

Renal proximal tubular epithelial cell line, HK-2, was obtained from American Type Culture Collection (ATCC, USA). The cells were cultured in Dulbecco’s Modified Eagle’s Medium (DMEM) containing 2 mM l-glutamine, 10 % fetal bovine serum (FBS) (Sigma Aldrich, USA) with supplemented with 1 % Penicillin (100 units/ml)-Streptomycin (10,000 lg/ml). The cells were maintained at condition of 37 °C and 5 % CO_2_. The cells were harvested when cell grown to sub-confluence and then cultured for 24 h in a medium containing 5.5 mmol/L glucose and 1 % FBS before being exposed to experimental conditions. For in vitro glucose incubating cell, the cells were divided into four groups. First group (Normal Glucose) treated with a low glucose concentration (5.5 mmol/L) was regarded as control. Cells in high glucose groups were supplemented with high concentrations of glucose (15, 30, 45 mM, respectively). The 45 mM glucose was chose to investigate the mechanism underlying high glucose induced cell injury. All cells were incubated with glucose for 48 h. To evaluate whether NF-κB regulates miR-29 expression in high glucose incubated HK-2 cell, the cells were pretreated with BAY11-7082 (5 μmol/L) (Sigma-Aldrich Co., St. Louis, MO, USA), an inhibitor for NF-κB pathway for 2 h before glucose treatment.

### Deacetylation analysis of Sirt1

Sirt1 acetylation activity detection kit (CY-1151, CycLex, Japan) was used in vitro assay of activity of Sirt1 deacetylation according to manufacturer’s instructions. In brief, total protein sample from tissue or cells were mixed with reaction containing 50 mmol/L Tris–HCl (pH 8.8), 0.5 mmol/L DTT, 0.25 mAU/ml Lysyl endopeptidase, 1 μmol/L Trichostatin A, 200 μmol/L NAD and 20 μmol/L fluoro-substrate peptide. The mixture was incubated for 60 min at 37 °C. Then the reacting solution was placed in Fluorescence Microplate (SpectraMax M2, USA) and fluorescence intensity was examined in light of 340 nm Emission Wavelength and 440 nm Excitation Wavelength. The final fluorescence value was normalized to protein concentration (fu/μg).

### NF-кB luciferase reporter assay

Cells were cultured in 35 mm dishes for 24 h and then were transfected with NF-кB-luciferase reporter plasmid (Promega, Madison, WI, USA) using lipofectamine 2000 (Invitrogen, Carlsbad, CA, USA) according to the manufacturers’ protocol. After a period of 6 h transfection, cells were divided into equivalent aliquots for different experimental treatment and seeded in 24-well plates. After 18 h incubation under condition of 37 °C in a 5 % CO_2_ incubator, cells were harvested for following glucose stimulation. Relative light units were measured using Fujifilm LAS3000 Imaging system. The luciferase activities were normalized to total protein contents and expressed as the fold increase relative to the activity of untreated controls.

### Quantitative real-time RT-PCR

Next, total RNA was from cells or tissues isolated used a phenol–chloroform extraction protocol and the purity and concentration of RNAs were evaluated with spectrophotometric analysis. RNA was transcribed with SuperScript III Reverse Transcriptase (Invitrogen, Carlsbad, CA, USA) using an oligo dT primer. Quantitative RT-PCR was performed on a Bio-Rad SYBR Green PCR Master Mix (Bio-Rad, Hercules, Calif., USA). The final volume of the PCR reaction mixture was 20 ml that contained 2 ml cDNA, 1 mM of each primer, 10 ml GoTaq qPCR Master Mix (Promega), and sterile water up to 20 ml. Calculated Keap1 mRNA expression levels were normalized to the expression levels of GAPDH and relative miR-29 level was normalized to U6 of the same cDNA sample. Relative quantification of gene expression was performed using the 2^−ΔΔCt^ calculations.

### Western blotting

Western blotting was used to evaluate nuclear Nrf2 expression and cytoplasmic p65 subunit of NF-κB and Keap1 expression. In Brief, samples were reconstituted in sample buffer and denatured by boiling for 5 min. Samples containing 30 mg protein were loaded on 10 % SDS-PAGE (Bio-Rad, California, USA). The separated proteins were then electrophoretic transferred to polyvinylidene fluoride (PVDF) membrane (0.45 mm) (Millipore, Germany). Nonspecific protein was blocked by incubation with 5 % nonfat dry milk in TBS for 2 h. Then membrane was blotted by rabbit polyclonal antibodies against acetyl-lysine p65 (1:1000; Thermo Fisher Scientific, USA), Keap1 (1:1000; Abcam, USA), GST (1:500; Santa Cruz biotechnology, CA, USA), NQO1 (1:500; Santa Cruz biotechnology, CA, USA) and Nrf2 (1:1000; Abcam, USA) for 16 h at 4 °C. β-actin was used as loading control for cytoplasmic proteins and α-tubulin was used as loading control for nuclear protein. The target protein was recognized by peroxidase-conjugated secondary antibodies (Monoclonal anti-rat IgG- peroxidase antibody; 1:2000, Sigma Co., USA) by 2 h incubation at room temperature. The bands were presented by reacting with Chemiluminescence (ECL). Relative protein band density was quantified by image J.

### Transfection

To achieve alteration of expression of miR-29, cells were treated with miR-29 mimic or inhibitor with the negative control (NC) of nonsense strand using Lipofectamine (Invitrogen). In brief, cells were seeded in 35 mm dishes at a density of 10^6^ per dish overnight before the transfection. Cells and lipofectamine 2000 were separately diluted with 50 µl Opti-MEM^®^. These two dilutions were then mixed followed with being incubated under condition of 5 % CO2 at 37 °C for 48 h. After incubation, the mixed medium was replaced with fresh medium and cells were harvest for 48 h of glucose stimulation. Finally, cell viability was assessed using MTT assay and nuclear extract, whole cell lysate and total RNA of cells were separately isolated for molecular measurement.

Ectopic expression of Sirt1 and p65 in HK-2 cells was realized through pcDNA-Sirt1 and pcDNA-p65 transfection, respectively. The sequences of Sirt1 and p65 were synthesized and cloned into the pCDNA3.1 (Invitrogen, Shanghai, China) vector. The empty pcDNA3.1 vector was used as a control. The prepared plasmid vector (pcDNA-Sirt1, pcDNA-p65 and pCDNA3.1) were transfected into HK-2 cell using Lipofectamine (Invitrogen) following the manufacturer’s protocol. Forty-eight hours post-transfection, the expression levels of Sirt1 and p65 were detected by western blotting.

### Luciferase reporter gene assay

HK-2 cells were seeded at a density of 2 × 10^4^ cells in 96-well plates. One day later, cells were co-transfected with pGL4-TK-Luc-Keap1 (0.5 μg) with control of pGL-SV40 (internal control, 0.018 μg) and miR-29 mimic or inhibitor. Forty-eight hours after transfection, cells were harvested, lysed, and analyzed for luminescence with the dual-luciferase assay system (Promega). Luciferase activity was normalized to pRL-SV40 activity. Experiments were repeated at least three times.

### Chip assay

The ChIP assay was carried out to detect the possible target miR-29 gene by p65. In brief, cells were cross-linked with 1 % formaldehyde followed by being broken using ultrasonication. Soluble chromatin was collected by 10,000*g* centrifugalization at 4 °C for 10 min and incubated with antibodies against p65 (Santa Cruz Biotechnology, Inc.) overnight at 4 °C for immunoprecipitation with rabbit IgG as negative control. The immune complex was washed and DNA sample was obtained by Gel Extraction Kit (Omega Bio-tek, GA, USA). The recovered DNA was then analyzed by qRT-PCR using primers flanking the ARE of the human miR-29 promoter. Results were normalized to input controls.

### In vitro ubiquitination assay

The procedures for the ubiquitin ligase activity assay were performed. Briefly, HK-2 cells were co-transfected with plasmids expressing Myc-tagged CUL3, T7-tagged KEAP1, and HA-tagged ROC1. Twenty-four hours after transfection, cells were lysed and immunoprecipitated with an anti-Myc antibody. Myc-CUL3 immunocomplexes immobilized on protein A-agarose beads were incubated with 1 μg of purified His-FLAG-tagged Nrf2 in a ubiquitin ligation reaction mixture (final volume, 30 μl) containing 50 mM Tris–HCl (pH 7.4), 5 mM MgCl2, 2 mM NaF, 10 nM okadaic acid, 2 mM ATP, 0.6 mM DTT, 1 μg of His6-tagged ubiquitin, 60 ng of E1 (Boston Biochem), and 300 ng of E2-UbcH5c at 37 °C for 1 h. The reaction was terminated by boiling for 5 min in a SDS sample buffer containing 0.1 M DTT, and the proteins were resolved by SDS-PAGE, followed by immunoblotting with an anti-FLAG antibody.

BMDCs cells were pre-cultured in a 100 mm dish for 24 h. Cells were supplemented with different treatments for another 12 h and then lysed with RIPA buffer containing proteinase inhibitor. The lysates were homogenized in an ultrasonicator for 10 s twice and incubated on ice for 30 min; the homogenates were then centrifuged at 15,000×*g* for 15 min. The concentration of protein extracts was detected by the Bradford method using a protein assay kit (Beyotime, China).

Immunoprecipitation and immunoblotting were conducted by conventional methods. In brief, the whole cell lysates with 0.5 mg of proteins were pretreated with protein A-Sepharose beads for 1 h, cultured with 1 μg anti-Keap1 or anti-Nrf2 antibody for 4 h to make Nrf2 and Keap1 immunoprecipitate. The immunoprecipitated complexes were then washed five times with RIPA buffer and boiled in SDS sample buffer for 5 min. The immunoprecipitation products were run on 8 % SDS-PAGE and electrophoretically transferred to a PVDF membrane (Bio-Rad, USA). These were then incubated with primary antibodies (Cell Signaling, USA) according to the manufacturer’s protocol, before being incubated with horseradish peroxidase conjugated secondary antibody for 2 h. The protein was finally detected and imaged using a GS800 Densitometer Scanner (Bio-Rad, USA).

### Statistical analysis

Results were expressed as mean ± standard deviation (SD). Analysis of variance (ANOVA) was used for multiple comparisons among groups followed by Tukey–Kramer post hoc analysis and student *t* test was used for evaluated difference change between two groups using the SPSS 16 statistical software. Results were considered significant at p < 0.05. To assess the correlation between the serum creatinine or blood urea nitrogen (BUN) and serum miR-29 level, the Spearman correlation coefficient was used. The relationship was described as the correlation coefficient r and statistical significance was defined as P < 0.05.

## Results

### General measurements in diabetic rats

**Fig. 1:**
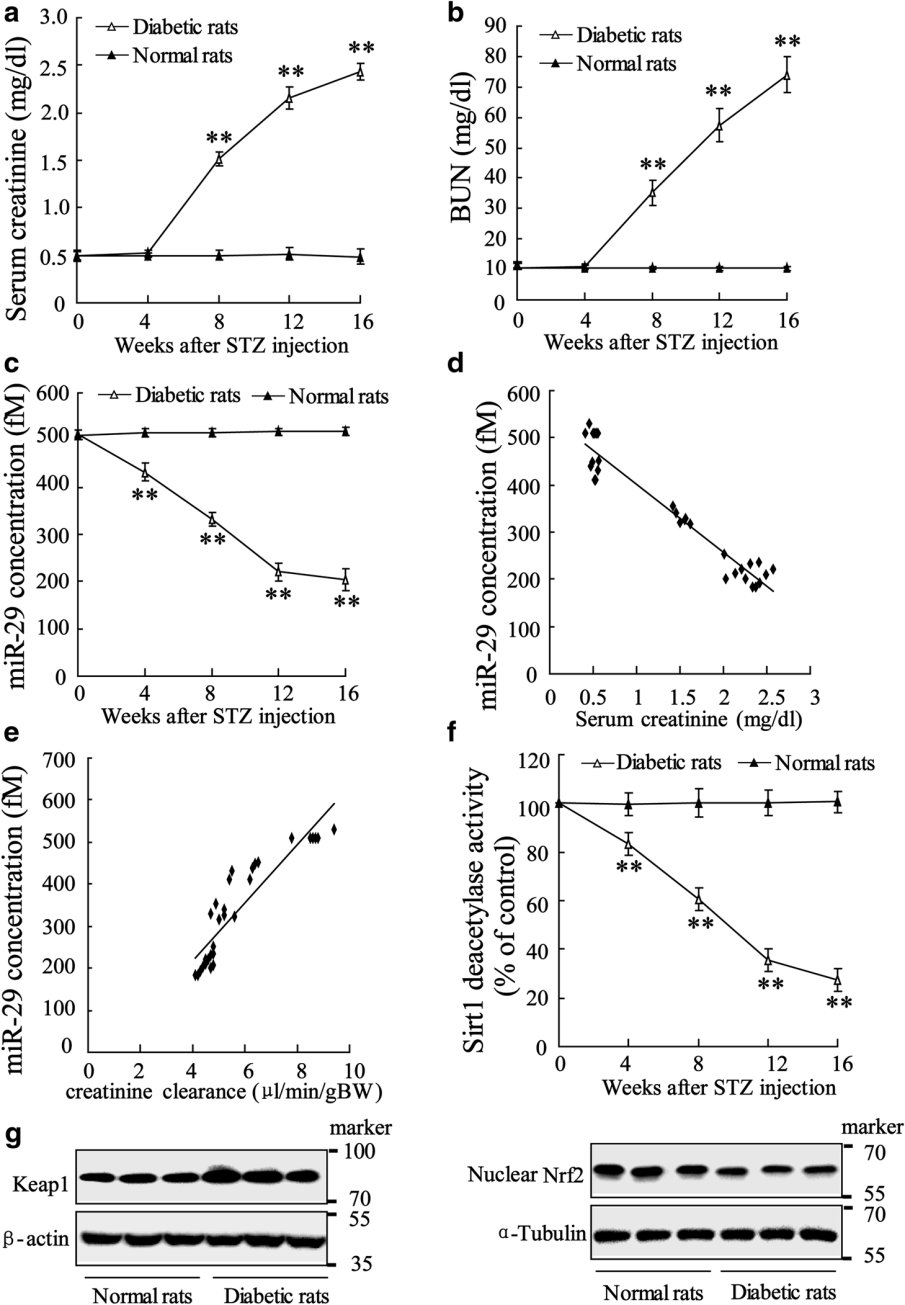
Correlation between blood parameters and signal molecules in diabetic rats. The rat model of diabetes was established by intraperitoneal injection of 65 mg/kg STZ. On week 0, 4, 8, 12, 16, **a** Serum creatinine and **b** blood urea nitrogen (BUN) were measured, and **c** renal tubule miR-29 level was determined using quantitative RT-PCR. Correlated analysis was performed to assess relationship between **d** miR-29 level and Serum creatinine as well as **e** miR-29 level and creatinine clearance. **f** Sirt1 activity was assessed. **g** Western blotting was performed to determine expressions of Keap1 and nuclear Nrf2 in isolated renal tubules. Data were presented as mean ± SD. **P < 0.01 vs. normal rats

As shown in Fig. 1a, b, compared with normal rat, levels of serum creatinine and blood urea nitrogen (BUN) were significantly elevated in diabetic rats at week of 8, 12 and 16 after STZ treatment. Renal tubule was obtained from rats treated with STZ for 4, 8, 12, 16 weeks and lyzed for analysis of miR-29 expression. The data showed that miR-29 level was decreased in time-dependent manner after rat model of diabetes established (Fig. 1c). The correlation analysis demonstrated that abnormal miR-29 expression is negatively related to serum creatinine (Spearman correlation is −0.96, P = 0.000; Fig. 1d) and creatinine clearance (Spearman correlation is −0.93, P = 0.000; Fig. 1e). These data indicated that miR-29 is involved in pathological process of diabetes and might function as one of modulatory factors. Additionally, we also observed that Sirt1 deacetylases activity was attenuated in diabetic rat and declined by the time (Fig. 1f). Diabetic rats expressed higher level of Keap1 and lower level of Nuclear Nrf2 in renal tubules (Fig. 1g). Additional file 1: Figure S1 presented that diabetic rats were with injured renal tubules indicated by vacuolar degeneration and extensive loss of brush border.

### General measurements in high glucose-triggered HK-2 cells

**Fig. 2:**
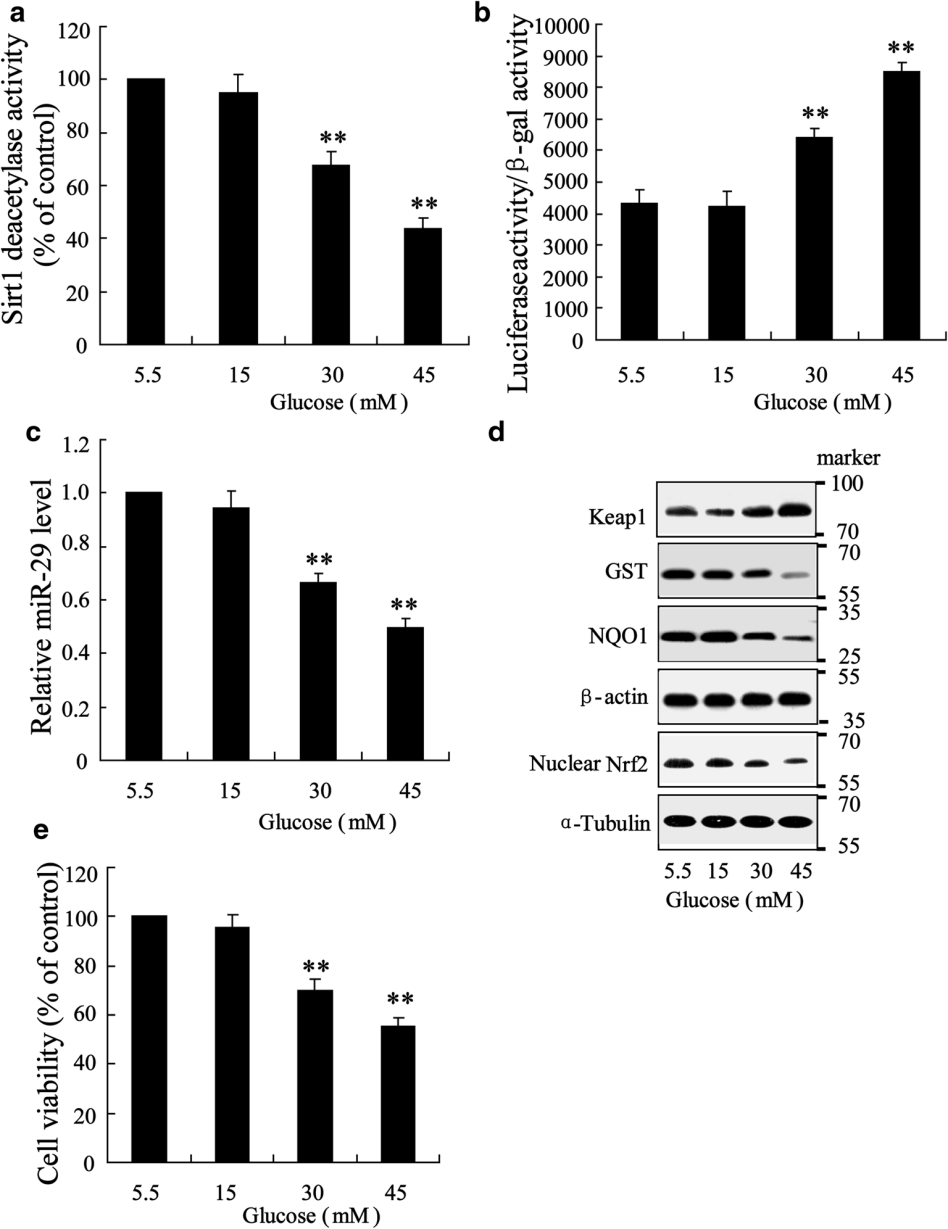
Effect of high glucose on renal tubule epithelia cell of HK-2 in vitro. Cells were triggered with doses of glucose (5.5, 15, 30 and 45) for 48 h. **a** Sirt1 activity was assessed. **b** NF-κB transcription activity was evaluated using luciferase reporter gene assay. **c** miR-29 expression was determined. **d** Western blot was performed to assess Keap1, GST, NQO1 and nuclear Nrf-2 expression. **e** Cell viability was evaluated using MTT assay. Data were presented as mean ± S.D. **P < 0.01 vs. cell treated with 5.5 mM glucose

To investigate the role of miR-29 in injured renal tubule of diabetes, the in vitro experimental protocols were performed in high glucose-triggered renal epithelia cell line, HK2. Cells were incubated with normal glucose (5.5 mM) and high glucose (15, 30, 45 mM) for 48 h and lyzed for analysis. We observed that Sirt1 deacetylases activity was declined as the increment of high glucose concentration (Fig. 2a). The transcriptional activity of NF-κB was increased with increasing of high glucose concentration (Fig. 2b). Level of miR-29 was significantly downregulated in cells incubated with 30 and 45 mM high glucose compared with cell treated with 5.5 mM normal glucose (Fig. 2c). In resul t of western blotting, we observed that expression of Keap1 was enhanced and expressions of GST, NQO1 and nuclear Nrf2 was deceased with increasing of glucose concentration (Fig. 2d). Treatment of 30 and 40 mM high glucose also substantially reduced cell viability (Fig. 2e).

### Overexpression of Sirt1 in high glucose-stimulated HK-2 cells

**Fig. 3:**
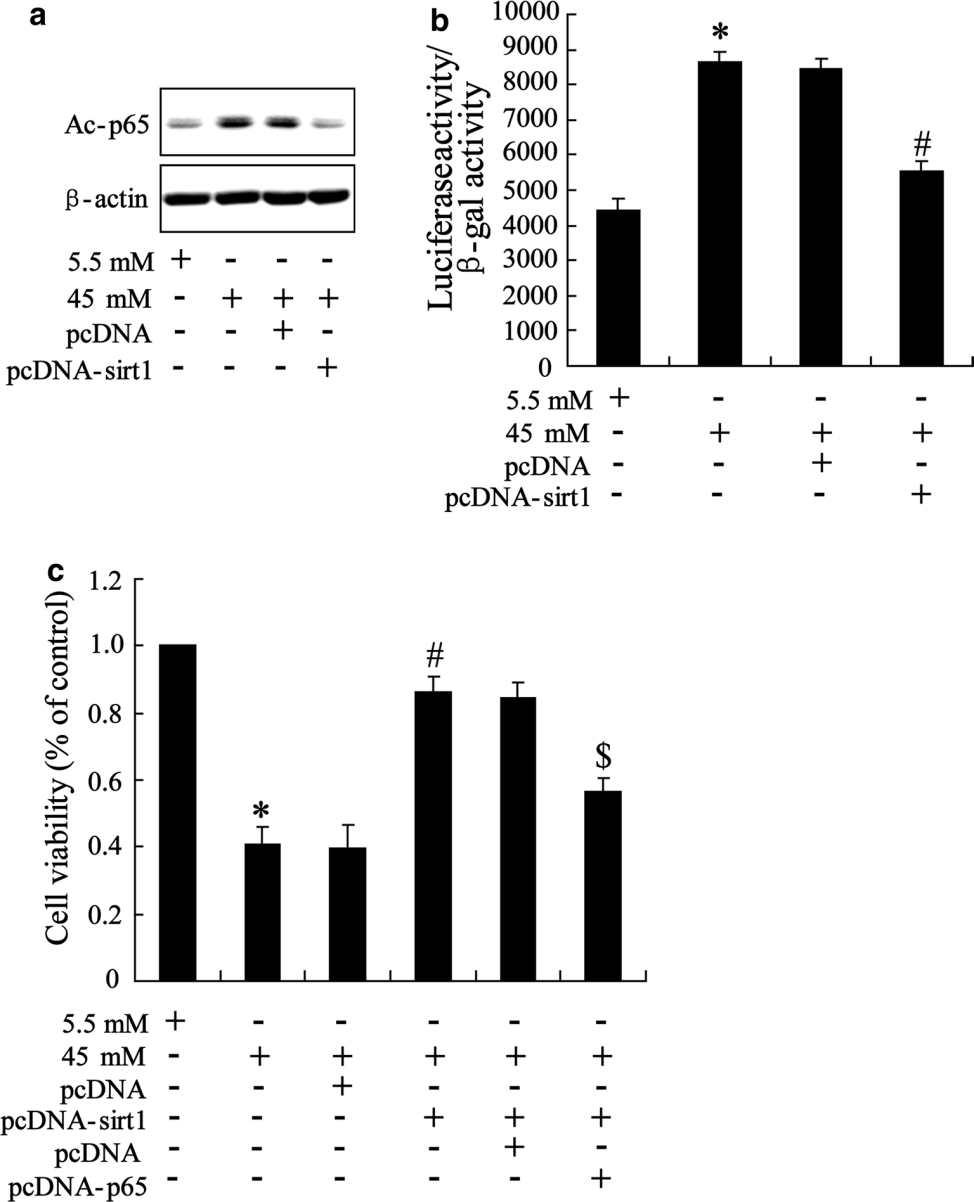
Effect of overexpression of Sirt1 on high glucose-stimulated HK-2 cells. Cells were pre-treated with pcDNA-Sirt1 with pcDNA as control. 48 h after incubation, cells were triggered with 5.5 mM and 45 mM glucose for 48 h. **a** Level of p65 acetylation, **b** translational activity of NF-κB and **c** cell viability were determined. Data were presented as mean ± SD. *P < 0.05 vs. cell treated with 5.5 mM glucose, ^#^P < 0.05 vs. cell treated with 45 mM + pcDNA

To assess the function of Sirt1 in high glucose-triggered HK-2 cell, we overexpressed the Sirt1 protein by pcDNA-Sirt1 transfection and determined the effect of Sirt1 on NF-κB activity and cell viability. The 45 mM high glucose was chose as final experimental concentration. As shown in Fig. 3a, overexpression of Sirt1 reversed high glucose-induced increase of acetyl-p65 expression. By luciferase reporter gene assay, we observed that high glucose-induced translational activity of NF-κB was abrogated by Sirt1 overexpression (Fig. 3b). These data suggested that Sirt1 regulating NF-κB activity is involved in process of high glucose-stimulating HK-2 cell. This was further confirmed by result of MTT assay which demonstrated that overexpression of Sirt1 improved high glucose-reduced cell viability while this action was reversed by pcDNA-65 transfection (Fig. 3c).

### NF-κB binds to miR-29

**Fig. 4:**
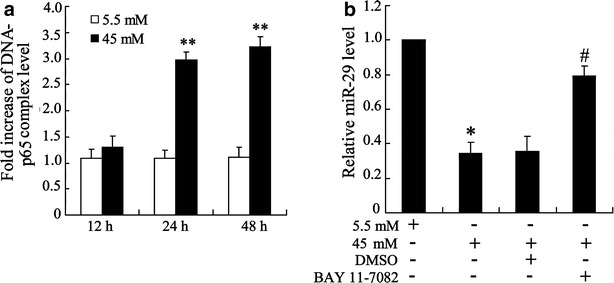
NF-κB regulates miR-29 expression in high glucose-triggered HK-2 cells. **a** Cells were stimulated with 5.5 and 45 mM glucose for 12, 24, 48 h and combination of p65 and miR-29 gene was examined using Chip assay. **b** Cells were exposed to BAY 11-7082 for 2 h before 5.5 and 45 mM glucose treatment and miR-29 expression was determined. Data were presented as mean ± S.D. *P < 0.05, **P < 0.01 vs. cell treated with 5.5 mM glucose, ^#^P < 0.05 vs. cell treated with 45 mM + DMSO

Chip assay was performed to assess whether NF-κB directly binds to miR-29 gene in high glucose cultured HK-2 cell. As shown in Fig. 4a, high glucose promoted association between p65 and miR-29 promotor in a manner of time dependent. This data suggested an expressional regulation of miR-29 by NF-κB. This was further confirmed by qRT-PCR assay which showed that inhibition of NF-κB pathway by BAY 11-7082 treatment increased miR-29 expression in high glucose-triggered cell.

### MiR-29 directly regulates Keap1

**Fig. 5:**
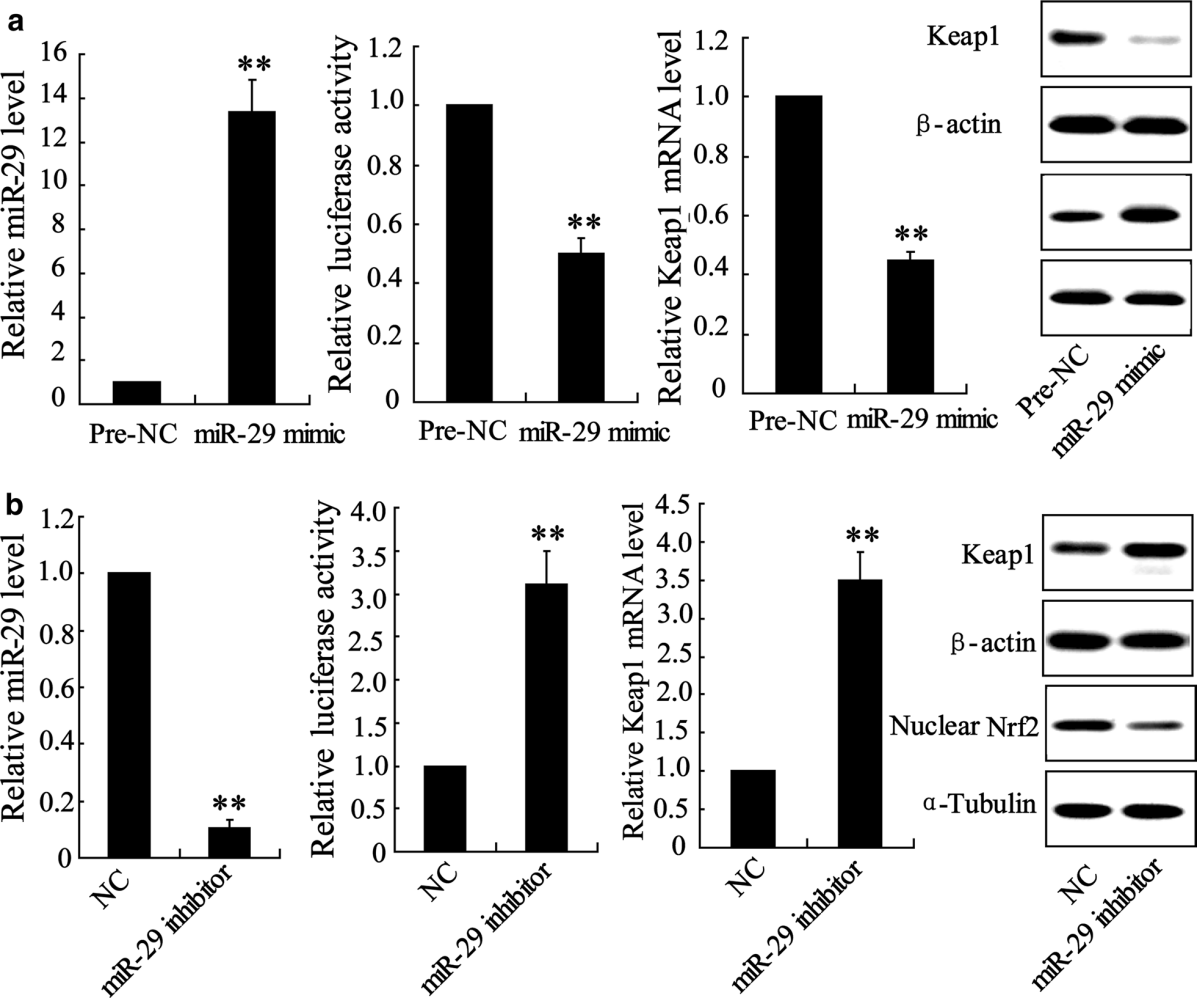
miR-29 regulates Keap1 expression in HK-2 cells. **a** Cells were transfected with miR-29 mimic and **b** miR-29 inhibitor as well as respective negative control (NC) for 48 h; miR-29 level, translational activity of Keap1 and expression of Keap1 mRNA and protein were determined. Data were presented as mean ± S.D. **P < 0.01 vs. pre-NC or NC

Detection of abnormal expression of miR-29 and Keap1 in HK-2 cell lead us to determine whether miR-29 directly regulates Keap1 expression. We altered miR-29 expression by cell incubated with miR-29 mimic or inhibitor. In HK-2^miR−29 mimic^ cell, level of miR-29 was significantly increased by 12.9 fold, binding activity of miR-29 and Keap1 promotor was reduced and expression of Keap1 mRNA and protein was attenuated compared with pre-NC incubated cell (Fig. 5a). The opposite trend occurred in HK-2^inhbitor^ cell. As shown in Fig. 5b, level of miR-29 declined by 10 fold, binding activity of miR-29 and Keap1 promotor was boosted that leads to increase of the expression of Keap1 mRNA and protein.

### High glucose promotes ubiquitination of Nrf2 via miR-29/Keap1 axis

**Fig. 6:**
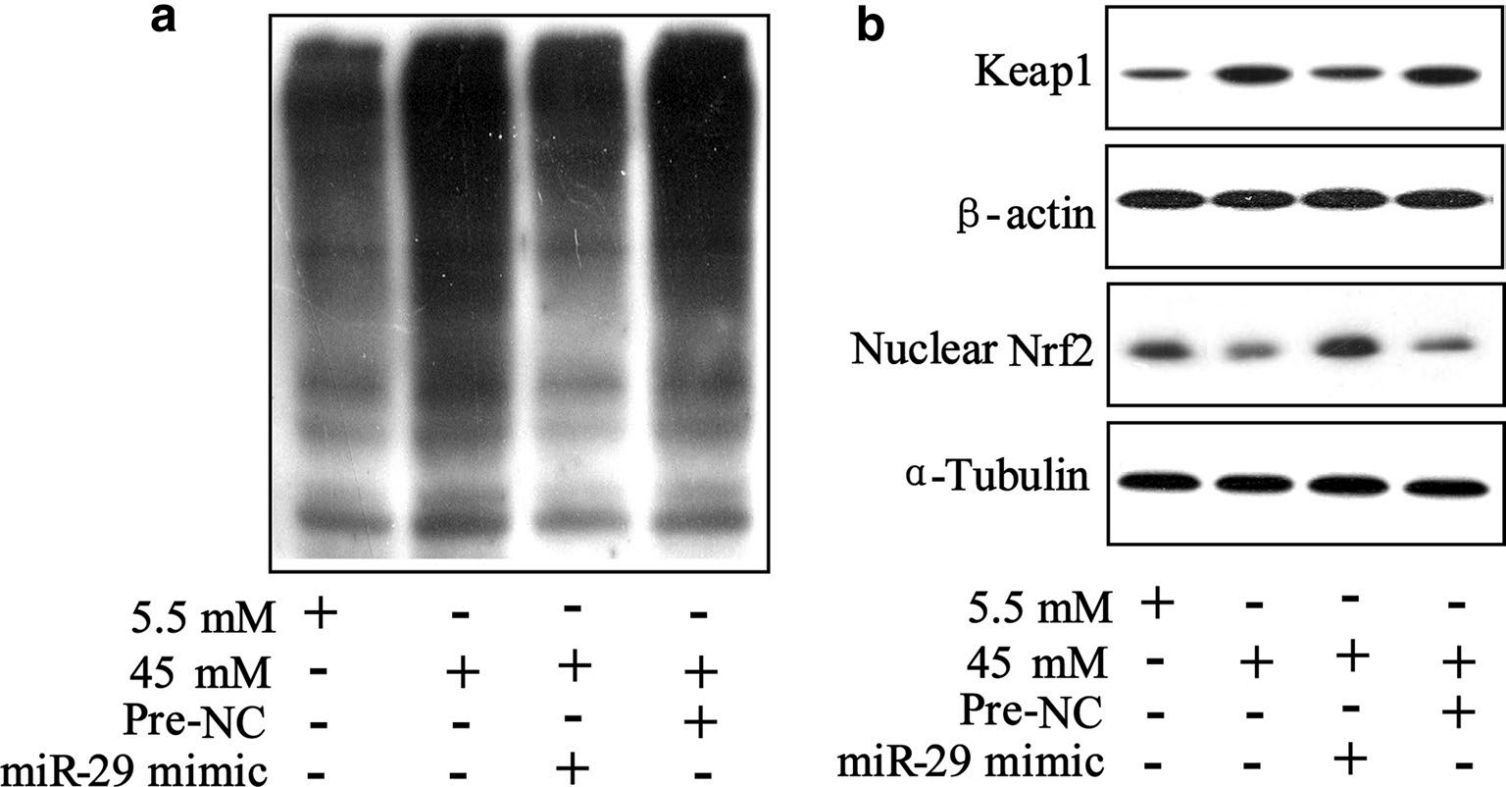
Ubiquitination of Nrf2 was regulated by miR-29/Keap1 axis in high glucose triggered HK-2 cells. **a** HK-2^miR−29mimic^ cells were transfected with Flag-ubiquitin and myc-Keap1 for 2 h and exposed to 5.5 and 45 mM glucose for 48 h; expression of ubiquitinated Keap1 protein was determined using western blot. **b** Western blot was performed to analyze expression of Keap1 and nuclear Nrf2 in 5.5 mM and 45 mM glucose-triggered HK-2^miR−29mimic^ cells

To determine the downstream molecule of miR-29/Keap1 axis, we examined Nrf2, a nuclear transcriptional factor that commonly activated by Keap1, in 45 mM high glucose-triggered HK-2^miR−29 mimic^ cell. The data showed that high glucose promoted ubiquitination of Nrf2 whereas this promotion was reversed by overexpression of miR-29 (Fig. 6a). By result of western blotting, we observed that high glucose induced upregulation of Keap1 and downregulation of nuclear Nrf2 was abrogated by miR-29 mimic treatment (Fig. 6b).

### Overexpression of miR-29 enhanced cell viability

To determine the role of miR-29 on high glucose triggered cell, cell viability was examined in HK-2^miR−29 mimic^ cell.

**Fig. 7:**
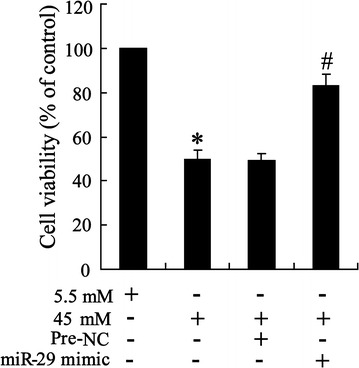
Effect of overexpression of miR-29 on cell viability in glucose-triggered HK-2 cell. Cells were transfected with miR-29 mimic and exposed to 5.5 and 45 mM glucose; cell viability was examined by MTT assay. Data were presented as mean ± S.D. *P < 0.05 vs. cell treated with 5.5 mM glucose, ^#^P < 0.05 vs. cell treated with 45 mM + Pre-NC

As shown in Fig. 7, reduction of cell viability by high glucose was effectively enhanced by miR-29 mimic treatment.

## Discussion

Diabetes mellitus is characteristic of metabolic disorders that contributed to multiple organ or tissue damage caused by complex systematic inflammatory and oxidative stress reactions [1]. At the best studied inflammatory mediated factor, NF-κB is rising as an important therapeutic target for precaution of diabetes induced kidney damage [17]. Thus much attention has been focused on the identification of downstream molecules of NF-κB. In this present study, we observed that NF-κB activity was enhanced mediated by high glucose induced reduction of deacetylases activity of Sirt1, which leaded to downregulation of miR-29 and inhibition of Keap/Nrf2 signal. Notably, both miR-29 and NF-κB activity play vital roles in high glucose induced decrease of cell viability. Our findings reveal a novel mechanism that modulates pathogenesis of renal tubular injury in response to high glucose.

Previous study points out that high glucose reduced Sirt1 expression that mediated protection against renal injury in diabetes [18]. This is also supported by our observation that overexpression of Sirt1 significantly reduced HK-2 cell viability. As a highly conserved NAD-dependent deacetylase that target histones, transcription factors, co-regulators, Sirt1 regulates adapt gene expression and metabolism in response to the cellular energy state [19]. The important role of Sirt1 in bodies’ activity is confirmed by a convincing proof which showed is a potential therapeutic target of Sirt1 for the treatment of the cellular metabolic memory [20]. Presently, accumulated evidence suggests that Sirt1 play a role in basal nuclear factor kappa B (NF-κB) repression via controlling its transcriptional silencing. Directly association between Sirt1 and p65 subunit of NF-kB plays an important role in high glucose-triggered human umbilical vein endothelial cells (HUVECs) [21]. Our study was in accordance with this previous study by providing evidence in which level of acetylated p65 was reduced by overexpression of Sirt1.

NF-κB is composed of a heterodimer of p50 and RelA/p65 subunits and its transcription factor signaling pathway is a key mediator of immune response in various diseases [22]. In unstimulated cells, NF-κB resides in the cytoplasm bound to its inhibitory proteins, which are members of the inhibitor of κB (IκB) family. While the p65 subunit would be liberated from NF-κB complex to translocate to the nucleus in response to stimulation of cells by environmental factors, including dietary fatty acids. Due to description of decreased expression of miR-29 by NF-κB signal in cells and tissues [12, 13], we speculated that NF-κB/miR-29 axis might be involved in high glucose incubated renal cell. This was demonstrated by the data of ChIP which showed a directly targeting miR-29 promotor by p65 and result of cells incubated NF-κB inhibitor, BAY 11-7082 that leaded to abolishment of high glucose-induced upregulation of miR-29 expression.

Anti-environmental press pathway serves as an important cellular defense against genotoxicity and oxidative stress, which has been observed to be closely correlated with dysregulation of blood glucose. Recently, the roles played by the Keap1-Nrf2 system, a famous anti-environmental press pathway, in the pathogenesis of diabetes mellitus and in the development of its complications have emerged as important research topics. For example, Nrf2 depletion increases renal oxidative and nitrosative stress in the streptozotocin (STZ)-induced mouse diabetes model [23]. Pathological analyses revealed that administration of Nrf2 inducers, i.e., sulforaphane and cinnamic aldehyde, suppressed STZ-mediated diabetic nephropathy [24]. Our data revealed that Nrf2 as well as its downstream molecules GST and NQO1 were downregulated while the expression of its regulator, Keap1 was upregulated in high glucose incubated HK-2 cell. These findings indicate that Nrf2 pathologically and functionally protects the kidney against diabetic damage. We next investigated whether miR-29 can serve as a regulatory element for Keap1/Nrf2 system. By luciferase reporter assays and expression analysis of Keap1 in HK^miR−29 mimic^ or HK^miR−29 inhibitor^ cells, we can draw a conclusion of miR-29 directly regulated Keap1 expression by targeting Keap1 mRNA 3′ UTR.

## Conclusion

In summary, our data suggested that high glucose induced renal tubular injury might be a process of a signal transduction pathway of Sirt1/NF-κB/miR-29/Keap1/Nrf2. This finding demonstrated a novel mechanism by which high glucose causes renal tubules injury and may provide insight as to what might be acted as therapy target by providing evidence supporting a role for miR-29 overexpression in the inhibition of Keap1/Nrf2 pathway.

## Additional file

10.1186/s12967-015-0710-y Histopathological analysis of renal tubules in normal rats and diabetic rats with hematoxylin and eosin (HE). Magnification 200×.
