# Supplementary figures and images for: High glucose induces renal tubular epithelial injury via Sirt1/NF-kappaB/microR-29/Keap1 signal pathway
Source: J Transl Med. 2015 Nov 9;13:352. doi: 10.1186/s12967-015-0710-y (PMC4640239; doi:10.1186/s12967-015-0710-y)

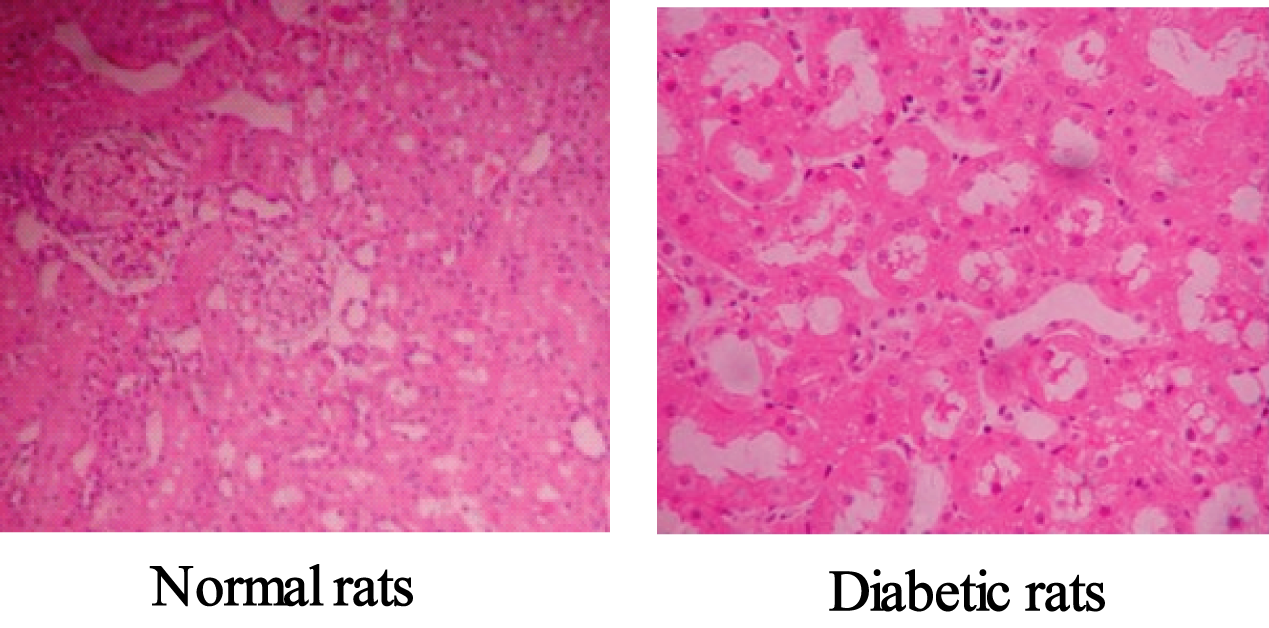

Supplement: Supplementary file 1 — 10.1186/s12967-015-0710-y Histopathological analysis of renal tubules in normal rats and diabetic rats with hematoxylin and eosin (HE). Magnification 200×. [file 12967_2015_710_MOESM1_ESM.tif]
